# Diammonium diaqua­bis(malonato-κ^2^ *O*,*O*′)cobaltate(II) dihydrate

**DOI:** 10.1107/S1600536808004625

**Published:** 2008-02-22

**Authors:** Haiyun Xu, Fengwu Wang

**Affiliations:** a Department of Chemistry, Huainan Normal College, 232001 Huainan, Anhui, People’s Republic of China

## Abstract

The title complex, (NH_4_)_2_[Co(C_3_H_3_O_4_)_2_(H_2_O)_2_]·2H_2_O, features a six-coordinate Co atom located on a center of symmetry. The octa­hedral O_6_ coordination geometry is defined by two bidentate malonate ligands and two water mol­ecules, with the latter in a *trans* configuration. The mol­ecules are linked through O—H⋯O and N—H⋯O hydrogen-bonding inter­actions, forming a three-dimensional supra­molecular network.

## Related literature

For related literature, see: Delgado *et al.* (2006 ▶); Saadeh *et al.* (1993 ▶); Wang *et al.* (2005 ▶); Wuest (2005 ▶); Yolanda *et al.* (2002 ▶).
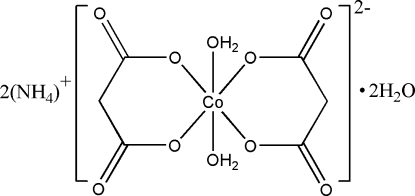


## Experimental

### 

#### Crystal data

(NH_4_)_2_[Co(C_3_H_3_O_4_)_2_(H_2_O)_2_]·2H_2_O
*M* *_r_* = 371.17
Triclinic, 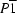

*a* = 6.950 (2) Å
*b* = 7.075 (2) Å
*c* = 7.433 (2) Å
α = 89.032 (5)°
β = 73.076 (5)°
γ = 88.062 (5)°
*V* = 349.45 (17) Å^3^
*Z* = 1
Mo *K*α radiation
μ = 1.29 mm^−1^
*T* = 298 (2) K
0.24 × 0.21 × 0.18 mm

#### Data collection

Bruker SMART APEX CCD diffractometer
Absorption correction: multi-scan (*SADABS*; Sheldrick, 1996 ▶) *T*_min_ = 0.747, *T*_max_ = 0.801
1817 measured reflections
1285 independent reflections
1246 reflections with *I* > 2σ(*I*)
*R*_int_ = 0.057

#### Refinement

*R*[*F* ^2^ > 2σ(*F* ^2^)] = 0.041
*wR*(*F* ^2^) = 0.107
*S* = 1.09
1285 reflections
97 parameters
4 restraints
H-atom parameters constrained
Δρ_max_ = 0.39 e Å^−3^
Δρ_min_ = −0.76 e Å^−3^

### 

Data collection: *SMART* (Siemens, 1996 ▶); cell refinement: *SAINT* (Siemens, 1996 ▶); data reduction: *SAINT*; program(s) used to solve structure: *SHELXS97* (Sheldrick, 2008 ▶); program(s) used to refine structure: *SHELXL97* (Sheldrick, 2008 ▶); molecular graphics: *SHELXTL* (Sheldrick, 2008 ▶); software used to prepare material for publication: *SHELXTL*.

## Supplementary Material

Crystal structure: contains datablocks global, I. DOI: 10.1107/S1600536808004625/tk2245sup1.cif

Structure factors: contains datablocks I. DOI: 10.1107/S1600536808004625/tk2245Isup2.hkl

Additional supplementary materials:  crystallographic information; 3D view; checkCIF report

## Figures and Tables

**Table 1:** Hydrogen-bond geometry (Å, °)

*D*—H⋯*A*	*D*—H	H⋯*A*	*D*⋯*A*	*D*—H⋯*A*
O5—H5*A*⋯O6^i^	0.85	1.90	2.723 (3)	164
O5—H5*B*⋯O4^i^	0.85	1.82	2.663 (3)	172
O6—H6*A*⋯O1^ii^	0.84	2.57	3.336 (3)	153
O6—H6*A*⋯O2^ii^	0.84	1.95	2.704 (3)	149
O6—H6*B*⋯O3^iii^	0.85	2.57	3.063 (3)	118
O6—H6*B*⋯O5^iii^	0.85	2.17	2.879 (3)	141
N1—H1*A*⋯O6^iv^	0.85	2.16	2.950 (3)	155
N1—H1*B*⋯O3^v^	0.85	1.97	2.805 (3)	165
N1—H1*C*⋯O4^vi^	0.85	2.33	2.988 (3)	135
N1—H1*D*⋯O2	0.85	2.06	2.857 (4)	155

Symmetry codes: (i) 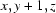; (ii) 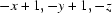; (iii) 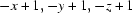; (iv) 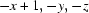; (v) 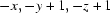; (vi) 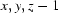.

**Fig. 1.**
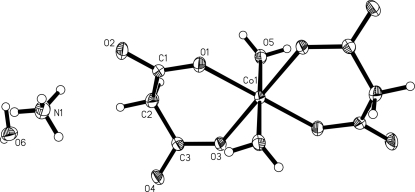
The structure of (I) expanded to show the coordination geometry of the Co atom which sits on a center of inversion; the unlabelled atoms are related by the symmetry operation -x, 2 - y, 1 - z. The figure shows 30% probability displacement ellipsoids and the atom-numbering scheme.

**Fig. 2.**
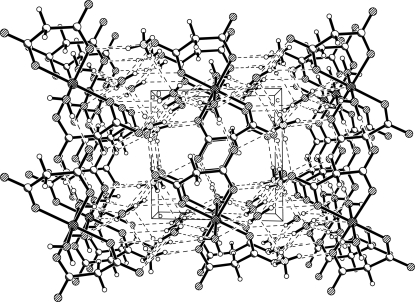
The 3-D superamolecular structure of (I). Hydrogen bond interactions are shown as dashed lines.

## supplementary crystallographic information

### Comment

In the design of supramolecular complexes, a well known and effective strategy is the matching of suitable hydrogen bond donors and acceptors (Wuest, 2005). Metal aqua-ions may act as excellent, readily available hydrogen bond donors with limited acceptor properties. Several novel complexes with metal aqua-ions have been reported (Delgado *et al.*, 2006; Saadeh *et al.*, 1993; Wang *et al.*, 2005; Yolanda *et al.*, 2002.) We report here the crystal structure of the title complex, (I), [NH_4_]_2_[Co(C_3_H_3_O_4_)_2_(OH_2_)_2_].2H_2_O, Fig. 1, in which the asymmetric comprises half a complex dianion, [Co(C_3_H_3_O_4_)_2_(OH_2_)_2_], situated on a center of inversion, an ammonium cation and a water molecule of crystallization.

The coordination polyhedron of the Co atom is that of an elongated octahedron defined by an O_6_ donor set. Four carboxylate O atoms, derived from two bidentate malonate ligands, build the equatorial plane, whereas two water molecules occupy the axial sites. As expected the Co—O_axial_ distance [2.1020 (19) Å] is longer than the Co—O_equatorial_ distances [2.0502 (18) and 2.0592 (17) Å]. The bond angles around the cobalt atom are close to that expected for an ideal octahedron. The molecules are linked through O—H···O and N—H···O hydrogen-bonding interactions and form a 3-D supramolecular network, Fig. 2 and Table 2.

### Experimental

Crystals of (I) were obtained by a diffusion method. In one arm of an U-tube was placed [NH_4_]_2_[C_3_H_2_O_4_] (30 mg, 0.2 mmol) in water/ethanol (1:1; 10 ml) and in the other [Co(ClO_4_)_2_].6H_2_O (37 mg, 0.1 mmol) in water/ethanol (1:1; 10 ml). The purple crystals were collected by filtration, washed with distilled water, followed by ethanol and dried under reduced pressure for 2 h. Analysis found: C 19.24, H 5.27, N 7.32; C_6_H_20_CoN_2_O_12_ requires: C 19.42, H 5.43, N 7.55.

### Refinement

All H atoms were placed geometrically with C—H, N—H and O—H distances of 0.97, 0.85 and 0.85 Å, respectively, and with *U*_iso_(H) = 1.2*U*_eq_(C, N, O). Hydroxyl-H atoms were allowed to rotate to best fit the experimental electron density.

### Crystal data

**Table:**

(NH_4_)_2_[Co(C_3_H_3_O_4_)_2_(H_2_O)_2_]·2H_2_O	*Z* = 1
*M**_r_* = 371.17	*F*_000_ = 193
Triclinic, *P*1	*D*_x_ = 1.764 Mg m^−^^3^
Hall symbol: -P 1	Mo *K*α radiation λ = 0.71073 Å
*a* = 6.950 (2) Å	Cell parameters from 1285 reflections
*b* = 7.075 (2) Å	θ = 2.9–25.5º
*c* = 7.433 (2) Å	µ = 1.29 mm^−^^1^
α = 89.032 (5)º	*T* = 298 (2) K
β = 73.076 (5)º	Block, purple
γ = 88.062 (5)º	0.24 × 0.21 × 0.18 mm
*V* = 349.45 (17) Å^3^	

### Data collection

**Table:**

Bruker SMART APEX CCD diffractometer	1285 independent reflections
Radiation source: fine-focus sealed tube	1246 reflections with *I* > 2σ(*I*)
Monochromator: graphite	*R*_int_ = 0.057
*T* = 298(2) K	θ_max_ = 25.5º
φ and ω scans	θ_min_ = 2.9º
Absorption correction: multi-scan(SADABS; Sheldrick, 1996)	*h* = −8→8
*T*_min_ = 0.747, *T*_max_ = 0.801	*k* = −7→8
1817 measured reflections	*l* = −6→8

### Refinement

**Table:**

Refinement on *F*^2^	Secondary atom site location: difference Fourier map
Least-squares matrix: full	Hydrogen site location: inferred from neighbouring sites
*R*[*F*^2^ > 2σ(*F*^2^)] = 0.041	H-atom parameters constrained
*wR*(*F*^2^) = 0.107	*w* = 1/[σ^2^(*F*_o_^2^) + (0.0668*P*)^2^ + 0.0816*P*] where *P* = (*F*_o_^2^ + 2*F*_c_^2^)/3
*S* = 1.09	(Δ/σ)_max_ < 0.001
1285 reflections	Δρ_max_ = 0.39 e Å^−^^3^
97 parameters	Δρ_min_ = −0.76 e Å^−^^3^
4 restraints	Extinction correction: none
Primary atom site location: structure-invariant direct methods	

### Special details

**Table:**

Geometry. All e.s.d.'s (except the e.s.d. in the dihedral angle between two l.s. planes) are estimated using the full covariance matrix. The cell e.s.d.'s are taken into account individually in the estimation of e.s.d.'s in distances, angles and torsion angles; correlations between e.s.d.'s in cell parameters are only used when they are defined by crystal symmetry. An approximate (isotropic) treatment of cell e.s.d.'s is used for estimating e.s.d.'s involving l.s. planes.
Refinement. Refinement of *F*^2^ against ALL reflections. The weighted *R*-factor *wR* and goodness of fit *S* are based on *F*^2^, conventional *R*-factors *R* are based on *F*, with *F* set to zero for negative *F*^2^. The threshold expression of *F*^2^ > σ(*F*^2^) is used only for calculating *R*-factors(gt) *etc*. and is not relevant to the choice of reflections for refinement. *R*-factors based on *F*^2^ are statistically about twice as large as those based on *F*, and *R*- factors based on ALL data will be even larger.

### Fractional atomic coordinates and isotropic or equivalent isotropic displacement parameters (Å^2^)

**Table:**

	*x*	*y*	*z*	*U*_iso_*/*U*_eq_	
Co1	0.0000	1.0000	0.5000	0.0246 (2)	
C1	0.2349 (4)	0.7294 (4)	0.2083 (4)	0.0302 (6)	
C2	0.3182 (4)	0.6385 (4)	0.3572 (4)	0.0371 (7)	
H2A	0.3696	0.5128	0.3143	0.045*	
H2B	0.4320	0.7108	0.3638	0.045*	
C3	0.1802 (4)	0.6198 (3)	0.5553 (3)	0.0265 (5)	
N1	0.1772 (4)	0.2806 (4)	0.0011 (3)	0.0438 (6)	
H1B	0.0825	0.2715	0.1031	0.053*	
H1A	0.2558	0.1845	−0.0309	0.053*	
H1C	0.1082	0.3123	−0.0726	0.053*	
H1D	0.2470	0.3754	0.0071	0.053*	
O1	0.1213 (3)	0.8738 (3)	0.2439 (2)	0.0322 (4)	
O2	0.2920 (4)	0.6563 (3)	0.0484 (3)	0.0501 (6)	
O3	0.0789 (3)	0.7649 (2)	0.6328 (2)	0.0315 (4)	
O4	0.1752 (3)	0.4670 (2)	0.6380 (3)	0.0371 (5)	
O5	0.2733 (3)	1.1242 (3)	0.4906 (3)	0.0347 (5)	
H5A	0.3694	1.1179	0.3893	0.042*	
H5B	0.2513	1.2374	0.5296	0.042*	
O6	0.6141 (3)	0.0563 (3)	0.2034 (3)	0.0391 (5)	
H6B	0.7011	0.0079	0.2521	0.047*	
H6A	0.6729	0.1133	0.1038	0.047*	

### Atomic displacement parameters (Å^2^)

**Table:**

	*U*^11^	*U*^22^	*U*^33^	*U*^12^	*U*^13^	*U*^23^
Co1	0.0283 (3)	0.0183 (3)	0.0245 (3)	0.00435 (19)	−0.0037 (2)	−0.00096 (19)
C1	0.0355 (14)	0.0214 (13)	0.0272 (13)	−0.0012 (11)	0.0012 (11)	−0.0005 (10)
C2	0.0345 (15)	0.0327 (15)	0.0364 (15)	0.0115 (12)	0.0003 (12)	0.0024 (12)
C3	0.0301 (13)	0.0227 (13)	0.0276 (13)	0.0007 (10)	−0.0103 (11)	−0.0007 (10)
N1	0.0573 (17)	0.0370 (14)	0.0322 (13)	0.0090 (12)	−0.0063 (12)	−0.0035 (10)
O1	0.0394 (11)	0.0270 (10)	0.0266 (9)	0.0088 (8)	−0.0047 (8)	−0.0021 (7)
O2	0.0817 (18)	0.0295 (11)	0.0280 (11)	0.0148 (11)	−0.0001 (11)	−0.0061 (8)
O3	0.0435 (11)	0.0218 (9)	0.0250 (9)	0.0067 (8)	−0.0040 (8)	0.0008 (7)
O4	0.0534 (13)	0.0203 (10)	0.0353 (11)	0.0045 (9)	−0.0099 (9)	0.0013 (8)
O5	0.0310 (10)	0.0241 (10)	0.0430 (11)	0.0009 (8)	−0.0012 (8)	−0.0054 (8)
O6	0.0400 (11)	0.0414 (12)	0.0322 (11)	−0.0001 (9)	−0.0054 (9)	0.0059 (9)

### Geometric parameters (Å, °)

**Table:**

Co1—O1	2.0502 (18)	C2—H2B	0.9699
Co1—O1^i^	2.0502 (18)	C3—O4	1.231 (3)
Co1—O3^i^	2.0592 (17)	C3—O3	1.272 (3)
Co1—O3	2.0592 (17)	N1—H1B	0.8500
Co1—O5^i^	2.1020 (19)	N1—H1A	0.8500
Co1—O5	2.1020 (19)	N1—H1C	0.8500
C1—O2	1.252 (3)	N1—H1D	0.8500
C1—O1	1.253 (3)	O5—H5A	0.8498
C1—C2	1.516 (4)	O5—H5B	0.8498
C2—C3	1.512 (4)	O6—H6B	0.8500
C2—H2A	0.9699	O6—H6A	0.8378
			
O1—Co1—O1^i^	180	C1—C2—H2A	107.8
O1—Co1—O3^i^	89.76 (7)	C3—C2—H2B	107.3
O1^i^—Co1—O3^i^	90.24 (7)	C1—C2—H2B	107.8
O1—Co1—O3	90.24 (7)	H2A—C2—H2B	107.1
O1^i^—Co1—O3	89.76 (7)	O4—C3—O3	122.4 (2)
O3^i^—Co1—O3	180	O4—C3—C2	119.0 (2)
O1—Co1—O5^i^	87.61 (8)	O3—C3—C2	118.6 (2)
O1^i^—Co1—O5^i^	92.39 (8)	H1B—N1—H1A	116.6
O3^i^—Co1—O5^i^	90.37 (8)	H1B—N1—H1C	99.2
O3—Co1—O5^i^	89.63 (8)	H1A—N1—H1C	116.0
O1—Co1—O5	92.39 (8)	H1B—N1—H1D	109.3
O1^i^—Co1—O5	87.61 (8)	H1A—N1—H1D	108.6
O3^i^—Co1—O5	89.63 (8)	H1C—N1—H1D	106.4
O3—Co1—O5	90.37 (8)	C1—O1—Co1	127.52 (17)
O5^i^—Co1—O5	180	C3—O3—Co1	127.00 (16)
O2—C1—O1	122.7 (3)	Co1—O5—H5A	118.7
O2—C1—C2	116.3 (2)	Co1—O5—H5B	109.9
O1—C1—C2	121.0 (2)	H5A—O5—H5B	111.0
C3—C2—C1	118.6 (2)	H6B—O6—H6A	109.3
C3—C2—H2A	107.7		

Symmetry codes: (i) −*x*, −*y*+2, −*z*+1.

### Hydrogen-bond geometry (Å, °)

**Table:**

*D*—H···*A*	*D*—H	H···*A*	*D*···*A*	*D*—H···*A*
O5—H5A···O6^ii^	0.85	1.90	2.723 (3)	164
O5—H5B···O4^ii^	0.85	1.82	2.663 (3)	172
O6—H6A···O1^iii^	0.84	2.57	3.336 (3)	153
O6—H6A···O2^iii^	0.84	1.95	2.704 (3)	149
O6—H6B···O3^iv^	0.85	2.57	3.063 (3)	118
O6—H6B···O5^iv^	0.85	2.17	2.879 (3)	141
N1—H1A···O6^v^	0.85	2.16	2.950 (3)	155
N1—H1B···O3^vi^	0.85	1.97	2.805 (3)	165
N1—H1C···O4^vii^	0.85	2.33	2.988 (3)	135
N1—H1D···O2	0.85	2.06	2.857 (4)	155

Symmetry codes: (ii) *x*, *y*+1, *z*; (iii) −*x*+1, −*y*+1, −*z*; (iv) −*x*+1, −*y*+1, −*z*+1; (v) −*x*+1, −*y*, −*z*; (vi) −*x*, −*y*+1, −*z*+1; (vii) *x*, *y*, *z*−1.
